# Validation of European Society of Cardiology pre-test probabilities for obstructive coronary artery disease in suspected stable angina

**DOI:** 10.1093/ehjqcco/qcaa006

**Published:** 2020-01-24

**Authors:** Rong Bing, Trisha Singh, Marc R Dweck, Nicholas L Mills, Michelle C Williams, Philip D Adamson, David E Newby

**Affiliations:** q1 BHF Centre for Cardiovascular Science, University of Edinburgh, 47 Little France Crescent, Edinburgh EH16 4TJ, UK; q2 Usher Institute of Population Health Sciences and Informatics, University of Edinburgh, 9 Little France Road, Edinburgh EH16 4UX, UK; q3 Christchurch Heart Institute, University of Otago, 2 Riccarton Avenue, Christchurch 8011, New Zealand

**Keywords:** Coronary artery disease, Pre-test probability, Computed tomography coronary angiography

## Abstract

**Aims:**

To assess contemporary pre-test probability estimates for obstructive coronary artery disease in patients with stable chest pain.

**Methods and results:**

In this substudy of a multicentre randomized controlled trial, we compared 2019 European Society of Cardiology (ESC)-endorsed pre-test probabilities with observed prevalence of obstructive coronary artery disease on computed tomography coronary angiography (CTCA). We assessed associations between pre-test probability, 5-year coronary heart disease death or non-fatal myocardial infarction and study intervention (standard care vs. CTCA). The study population consisted of 3755 patients (30–75 years, 46% women) with a median pre-test probability of 11% of whom 1622 (43%) had a pre-test probability of >15%. In those who underwent CTCA (*n* = 1613), the prevalence of obstructive disease was 22%. When divided into deciles of pre-test probability, the observed disease prevalence was similar but higher than the corresponding median pre-test probability [median difference 2.3 (1.3–5.6)%]. There were more clinical events in patients with a pre-test probability >15% compared to those at 5–15% and <5% (4.1%, 1.5%, and 1.4%, respectively, *P* < 0.001). Across the total cohort, fewer clinical events occurred in patients who underwent CTCA, with the greatest difference in those with a pre-test probability >15% (2.8% vs. 5.3%, log rank *P* = 0.01), although this interaction was not statistically significant on multivariable modelling.

**Conclusion:**

The updated 2019 ESC guideline pre-test probability recommendations tended to slightly underestimate disease prevalence in our cohort. Pre-test probability is a powerful predictor of future coronary events and helps select those who may derive the greatest absolute benefit from CTCA.

## Introduction

Chest pain is one of the commonest symptoms in patients presenting to the cardiology clinic. Determining whether obstructive coronary artery disease is the underlying cause requires a combination of clinical evaluation and, where appropriate, non-invasive or invasive investigations. However, additional testing should only be applied to an appropriately selected population, taking into account disease prevalence and the diagnostic performance of the test. Inappropriate testing may lead to under or over diagnosis of coronary artery disease, with potential misallocation of downstream invasive angiography and therapies. This is pertinent in the current era, where a broad range of functional and anatomical investigations across a spectrum of cost and availability can be performed. Furthermore, pre-test probabilities derived from historic cohorts have consistently overestimated contemporary disease prevalence,^1–4^ undoubtedly leading to over-testing.

The latest 2019 European Society of Cardiology (ESC) guidelines for chronic coronary syndromes^5^ have re-evaluated the assessment of patients with stable chest pain presenting to the outpatient cardiology clinic. The guideline has updated estimation of pre-test probability for obstructive coronary artery disease based on chest pain type, age, and gender using more contemporaneous data from the Coronary CT Angiography Evaluation For Clinical Outcomes: An International Multicenter (CONFIRM) Registry, the CT group of the Prospective Multicenter Imaging Study for Evaluation of Chest Pain (PROMISE) randomized controlled trial and a retrospective single-centre angiographic cohort.^1^^,^^3^^,^^4^^,^^6^ The thresholds that warrant further downstream testing have also been revised such that patients with a pre-test probability <5% are not recommended to undergo further testing, while testing is deemed appropriate in those with a pre-test probability >15%. Patients with an intermediate pre-test probability of 5–15% are recognized to have a good prognosis and testing may safely be deferred, although discretionary investigation can have a role. If testing is to be performed, the choice of test is determined by several factors, but in general, the guideline authors favour functional testing where the likelihood of obstructive disease is higher, and computed tomography coronary angiography (CTCA) where the likelihood is lower.

In this study, we assessed the latest ESC-endorsed pre-test probability estimates for obstructive coronary artery disease in the Scottish COmputed Tomgraphy of the HEART (SCOT-HEART, NCT01149590) trial population. We aimed to (i) validate the prediction of obstructive coronary artery disease by pre-test probability in our external cohort, (ii) assess the prognostic value of pre-test probability, and (iii) examine whether the use of CTCA was associated with a difference in clinical outcomes across pre-test probability strata.

## Methods

### Study population

This is a *post hoc* analysis of the open-label, randomized SCOT-HEART trial. The trial design, primary analysis, and 5-year outcomes have been reported previously.^7–9^ In brief, SCOT-HEART enrolled patients 18–75 years of age who were assessed in cardiology clinics with stable chest pain and randomized them to either routine care or routine care plus CTCA. Between November 2010 and September 2014, 4146 patients were recruited from 12 centres across the UK. Major exclusion criteria included severe chronic kidney disease (serum creatinine >200 μmol/L or estimated glomerular filtration rate <30 mL/min/1.73 m^2^) and acute coronary syndrome within 3 months. Ethical approval was provided by the South East Scotland Research Ethical Committee 02, Edinburgh, UK (10/S1102/43). To maintain consistency with the revised ESC guidelines, for this analysis we excluded patients with previously documented coronary heart disease and those <30 years of ages.

### Risk group assignment and endpoints

Patients were assigned a pre-test probability using chest pain type (typical, atypical, or non-anginal according to major society guidelines)^5^^,^^10^ age groups and gender according to ESC guidelines.^5^ Typical angina is defined as the following three criteria: (i) constricting discomfort in the chest, neck, jaw, shoulder or arm that is (ii) precipitated by exertion and (iii) relieved by rest or nitrates within 5 min. Atypical angina meets two of these criteria, while non-anginal symptoms meet one or none. Patients were then categorized as low (<5%), intermediate (5–15%), or high pre-test probability (>15%).

The presence of obstructive coronary artery disease was defined as coronary artery area stenosis >70% in a major epicardial vessel or >50% in the left main stem on CTCA as previously reported.^7^ The clinical endpoint was coronary heart disease death or non-fatal myocardial infarction at 5 years.^8^

### Statistical analysis

Continuous variables are reported as median [interquartile range, (IQR)] and were compared with the Kruskal–Wallis test. Categorical variables were compared with the *χ*^2^ test.

To validate the ESC pre-test probability estimates, patients were assigned a pre-test probability according to age, gender, and chest pain type and were compared with the observed prevalence in those patients who underwent CTCA. The study cohort was then divided into deciles according to pre-test probability. For each decile, median (IQR) pre-test probability and observed prevalence were determined.

For the clinical endpoint of non-fatal myocardial infarction or coronary heart disease death, cumulative event rates were examined using Kaplan–Meier curves and the log-rank test. Univariable and multivariable Cox proportional hazards models were constructed to determine clinical factors associated with the combined endpoint and to test the effect of CTCA with pre-test probability as an interaction. Variables included ESC pre-test probability category, study allocation and the remaining clinical variables used for minimization in the primary trial design (body mass index, diabetes mellitus, and atrial fibrillation). Two-sided *P*-values <0.05 were considered statistically significant. Analysis was performed using R version 3.5.0 (R Foundation for Statistical Computing, Vienna, Austria).

## Results

Of the 4146 patients enrolled in SCOT-HEART, 376 were excluded due to documented coronary heart disease (*n* = 372) or missing data for this field (*n* = 4) and 15 patients were <30 years of age, leaving a study population of 3755 (*Table 1*; Supplementary material online, *Figure S1*).

**Table 1:** Baseline characteristics stratified by pre-test probability

	Overall	<5%	5–15%	>15%	*P*
*n*	3755	831	1302	1622	
Female	1715 (45.7)	479 (57.6)	914 (70.2)	322 (19.9)	<0.001
Age (years)	57.0 (50.0–64.0)	47.0 (42.0–51.5)	56.0 (51.0–61.0)	63.0 (58.0–68.0)	<0.001
Chest pain type					<0.001
Non-anginal	1606 (42.8)	789 (94.9)	566 (43.5)	251 (15.5)	
Atypical angina	889 (23.7)	25 (3.0)	492 (37.8)	372 (22.9)	
Typical angina	1260 (33.6)	17 (2.0)	244 (18.7)	999 (61.6)	
Hypertension	1211 (32.5)	159 (19.3)	402 (31.2)	650 (40.3)	<0.001
Hyperlipidaemia	2078 (55.3)	299 (36.0)	662 (50.8)	1117 (68.9)	<0.001
Diabetes	370 (9.9)	58 (7.0)	123 (9.4)	189 (11.7)	0.001
Family history CAD	1549 (41.7)	376 (45.7)	606 (47.1)	567 (35.2)	<0.001
Smoking history					<0.001
Non-smoker	770 (20.5)	253 (30.4)	276 (21.2)	241 (14.9)	
Ex-smoker	1177 (31.4)	193 (23.2)	369 (28.3)	615 (38.0)	
Current smoker	1806 (48.1)	385 (46.3)	657 (50.5)	764 (47.2)	
Cerebrovascular disease	123 (3.3)	13 (1.6)	35 (2.7)	75 (4.6)	<0.001
Peripheral vascular disease	42 (1.1)	6 (0.7)	7 (0.5)	29 (1.8)	0.003
Systolic blood pressure (mmHg)	140.0 (125.0–152.0)	130.0 (120.0–145.0)	140.0 (125.0–152.0)	140.0 (130.0–157.0)	<0.001
Diastolic blood pressure (mmHg)	80.0 (76.0–90.0)	80.0 (75.0–89.0)	81.0 (76.0–90.0)	80.0 (77.0–90.0)	0.044
Body mass index (kg/m^2^)	28.8 (25.6–32.8)	29.1 (25.2–33.1)	29.0 (25.6–33.8)	28.5 (25.8–31.9)	0.029
Antiplatelet	1662 (44.3)	121 (14.6)	493 (37.9)	1048 (64.6)	<0.001
Statin	1459 (38.9)	103 (12.4)	405 (31.1)	951 (58.6)	<0.001
ACE inhibitor	497 (13.2)	66 (7.9)	143 (11.0)	288 (17.8)	<0.001
Beta-blocker	786 (20.9)	57 (6.9)	215 (16.5)	514 (31.7)	<0.001

CAD, coronary artery disease.

### Distribution of pre-test probabilities

**Figure 1:**
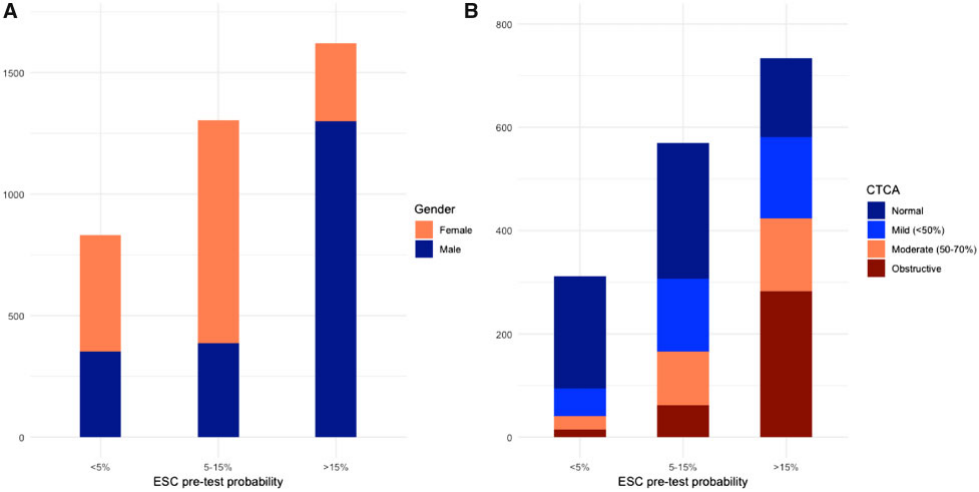
(*A*) Distribution of European Society of Cardiology pre-test probabilities in the study cohort, stratified by gender. (*B*) Prevalence of disease on computed tomography coronary angiography in each European Society of Cardiology group in patients who underwent computed tomography coronary angiography. CTCA, computed tomography coronary angiography; ESC, European Society of Cardiology.

Less than half the cohort had a pre-test probability of >15% (*n* = 1622, 43%). These patients were older and predominantly male (*n* = 1300, 80.1%) with a preponderance of typical symptoms. The intermediate group (pre-test probability 5–15%) were predominantly female (*n* = 914, 70.2%) (*Figure 1A*). Those not recommended for further investigation (pre-test probability <5%) had a more balanced gender distribution (*n* = 352, 42.4% males). There was a higher prevalence of cardiovascular risk factors and concomitant cardiovascular disease in the >15% group, including hypertension, hyperlipidaemia, diabetes mellitus, cerebrovascular disease, and peripheral vascular disease. Baseline preventative therapies increased with pre-test probability (*Table 1*, *P* < 0.001 for all).

### Pre-test probability and observed prevalence

**Figure 2:**
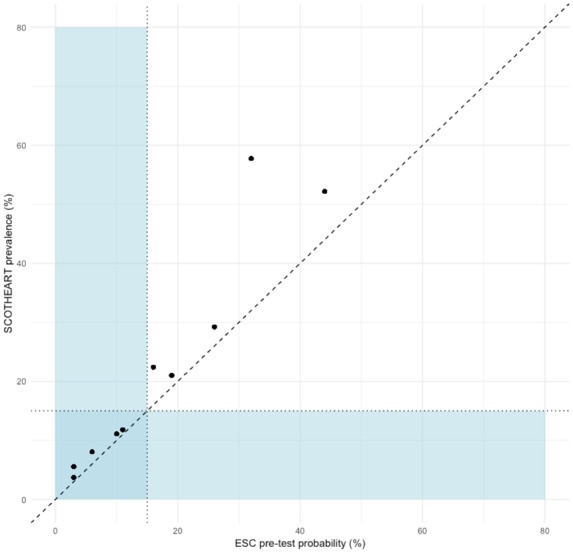
European Society of Cardiology pre-test probability vs. observed disease prevalence. Each point represents the median European Society of Cardiology pre-test probability per decile of the study population, plotted against the observed prevalence within each decile. The diagonal reference line represents perfect agreement. The shaded blue rectangles denote pre-test probabilities and prevalence of <15%—patients in whom testing may be safely deferred. ESC, European Society of Cardiology.

Amongst the 1613 patients who underwent CTCA, there was an increase in non-obstructive and obstructive disease across pre-test probability categories as well as an increase in coronary artery calcium score (*Figure 1B* and *Table 2*). There was a similar distribution of pre-test probabilities and observed prevalence of obstructive coronary artery disease in the CT cohort across patient categories (*Table 3*), with most groups having the same recommended strategy for further testing. The highest observed prevalence was in males >70 years with typical angina (61%)—the highest pre-test probability group (52%). Overall, men had a higher observed prevalence than women within each age and symptom strata, apart from non-anginal chest pain in those <30–39 years (where absolute numbers were low). All groups of women <70 years with atypical or non-anginal chest pain had both a pre-test probability and an observed prevalence of <15%. Deciles of pre-test probability and the observed prevalence within each decile are presented in *Figure 2* and Supplementary material online, *Figure S2*. Notably, five of the deciles had a median pre-test probability <15%, each with a corresponding median observed prevalence <15%. The observed prevalence of disease in each decile was similar but consistently higher than the corresponding median pre-test probability [median difference 2.3 (1.3–5.6)%].

**Table 2:** Computed tomography coronary angiography findings stratified by pre-test probability

	<5%	5–15%	>15%	*P*
*n*	311	569	733	
CTCA				<0.001
Normal	216 (69.5)	262 (46.0)	152 (20.7)	
Mild (<50%)	54 (17.4)	141 (24.8)	158 (21.6)	
Moderate (50–70%)	26 (8.4)	104 (18.3)	141 (19.2)	
Obstructive	15 (4.8)	62 (10.9)	282 (38.5)	
Calcium score (AU)	0.0 (0.0–3.0)	1.0 (0.0–47.0)	111.0 (6.0–444.0)	<0.001

AU, Agatston units; CTCA, computed tomography coronary angiography.

**Table 3:** Pre-test probabilities and observed prevalence of obstructive coronary artery disease in SCOT-HEART

	Typical		Atypical		Non-anginal	
	Male	Female	Male	Female	Male	Female
ESC 2013						
30–39	59%	28%	29%	10%	18%	5%
40–49	69%	37%	38%	14%	25%	8%
50–59	77%	47%	49%	20%	34%	12%
60–69	84%	58%	59%	28%	44%	17%
70+	89%	68%	69%	37%	54%	24%
ESC 2019						
30–39	3%	5%	4%	3%	1%	1%
40–49	22%	10%	10%	6%	3%	2%
50–59	32%	13%	17%	6%	11%	3%
60–69	44%	16%	26%	11%	22%	6%
70+	52%	27%	34%	19%	24%	10%
SCOT-HEART						
30–39	0%	0%	0%	0%	0%	13%
	*0/2*	*0/3*	*0/5*	*0/5*	*0/18*	*1/8*
40–49	30%	9%	17%	0%	6%	4%
	*9/30*	*3/33*	*7/41*	*0/44*	*6/107*	*2/53*
50–59	57%	18%	21%	12%	12%	5%
	*65/114*	*12/66*	*16/75*	*8/67*	*15/125*	*6/110*
60–69	53%	27%	37%	6%	17%	9%
	*77/146*	*24/89*	*19/52*	*4/67*	*16/92*	*9/103*
70+	61%	30%	54%	21%	33%	17%
	*28/46*	*11/37*	*7/13*	*5/24*	*5/15*	*4/23*

Grey shading denotes a pre-test probability of 5–15% (intermediate likelihood) in whom discretionary testing is reasonable. Green shading denotes a pre-test probability >15%, in whom further investigation is recommended.

ESC, European Society of Cardiology; SCOT-HEART, Scottish COmputed Tomography of the HEART.

For all ESC categories of patients with a pre-test probability >15%, there was a corresponding observed prevalence of >15%. Conversely, three groups of patients (*n* = 130, 8%) had a pre-test probability between 5% and 15% but an observed prevalence >15%. Two-thirds of these patients were female (*n* = 89). Of the female patients, 66 had typical angina and 23 had non-anginal symptoms, with a median age of 57 (53–70 years). In contrast, the 41 males were from the group aged 40–49 [median 45 (43–47)] years with atypical angina.

### Pre-test probability, outcomes, and study allocation

**Figure 3:**
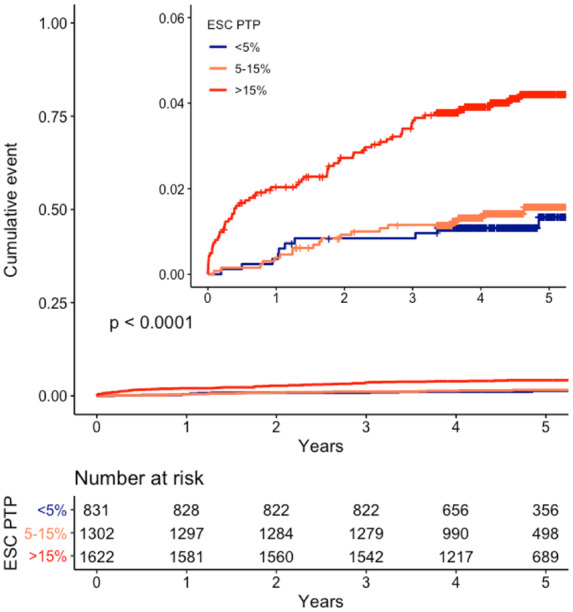
Cumulative incidence of coronary heart disease death or non-fatal myocardial infarction stratified by European Society of Cardiology pre-test probability categories. ESC, European Society of Cardiology; PTP, pre-test probability.

The combined endpoint of death from coronary heart disease or non-fatal myocardial infarction was met in 66 of 1622 (4.1%) patients with a high pre-test probability, 19 of 1302 with intermediate pre-test probability (1.5%), and 12 of 831 (1.4%) with low pre-test probability (*Figure 3*, *P* < 0.001 between groups).

**Figure 4:**
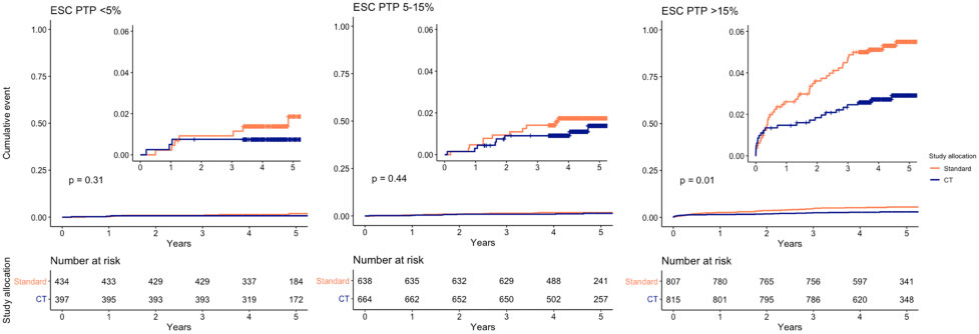
Cumulative incidence of coronary heart disease death or non-fatal myocardial infarction for each European Society of Cardiology pre-test probability category, stratified by study allocation. ESC, European Society of Cardiology; PTP, pre-test probability.

Events were further stratified within each group according to study arm allocation (CT or standard care; *Figure 4* and *Table 4*). There was more change in preventative medical therapies with the use of CTCA (Supplementary material online, *Table S1*). There were numerically fewer events in those patients who were randomized to CTCA, which was most evident in the high pre-test probability group [43 of 807 (5.3%) standard care vs. 23 of 815 (2.8%) CTCA, *P* = 0.01]. Within this group, there appeared to be fewer events in the CTCA arm regardless of whether patients were above or below the median pre-test probability (26%) (Supplementary material online, *Figure S3* and Supplementary material online, *Table S2*). On Cox regression analysis, a high pre-test probability was a strong predictor of the combined endpoint on both univariable analysis and multivariable analysis (hazard ratio 2.92, 95% confidence interval 1.37–6.20, *P* = 0.006). The use of CTCA was associated with a lower risk of the combined endpoint on univariable but not multivariable analysis, while there was no significant interaction between pre-test probability and the use of CTCA with respect to clinical outcomes (*P*_interaction_ 0.75 and 0.95 for intermediate and high pre-test probability, respectively; Supplementary material online, *Table S3*).

**Table 4:** Clinical outcomes

Pre-test probability	Standard care	CTCA	*P*
<5%	434	397	
** **NFMI/CHD death	8 (1.8)	4 (1.0)	0.31
** **NFMI	7 (1.6)	3 (0.8)	
** **CHD death	1 (0.2)	1 (0.3)	
5–15%	638	664	
** **NFMI/CHD death	11 (1.7)	8 (1.2)	0.44
** **NFMI	11 (1.7)	8 (1.2)	
** **CHD death	0	0	
>15%	807	815	
** **NFMI/CHD death	43 (5.3)	23 (2.8)	0.01
** **NFMI	39 (4.8)	21 (2.6)	
** **CHD death	5 (0.6)	2 (0.2)	

CHD, coronary heart disease; CTCA, computed tomography coronary angiography; NFMI, non-fatal myocardial infarction.

## Discussion

In this study, we demonstrate that the 2019 ESC estimates of pre-test probability for obstructive coronary artery disease in suspected stable angina are broadly similar to the observed prevalence in the SCOT-HEART trial cohort, although it now tends to underestimate prevalence. In this representative and general population of patients referred with stable chest pain to a cardiology clinic, less than half of patients would need further testing according to the current ESC guidelines. Furthermore, we have confirmed the strong prognostic value of pre-test probability and shown that CTCA may be most beneficial in those with a pre-test probability >15%.

Major society guidelines are an integral part of modern clinical medicine. As expert consensus documents, they provide a framework for best evidence-based practice within the limits of available data. It is, however, important for clinicians to tailor their approach on an individual basis, taking into account factors such as patient preferences, local resources, and differences between the patient at hand and derivation populations used to formulate guidelines. It has been established across several cohort studies that the previous ESC Diamond and Forrester pre-test probabilities overestimated the prevalence of disease, with a previous collaboration between SCOT-HEART and PROMISE investigators demonstrating more identification of low-risk patients with the symptom-based National Institute of Health and Care Excellence guidelines.^11^ Indeed, although not the focus of this analysis, the Diamond and Forrester estimates exceeded the observed disease prevalence in nearly all groups in our cohort (*Table 3*). This led to changes in the most recent iteration of the ESC guidelines^5^^,^^12^ which pooled three separate cohorts for this update. It is important to understand the patient populations, definitions, and potential biases used in these derivation cohorts, two of which were registries of consecutive patients and the third of which was a randomized controlled trial. Cheng *et al*.^1^ comprised 14 048 consecutive patients from the CONFIRM registry from Canada, Italy, South Korea, Switzerland, Germany, and the USA. The presence of coronary artery disease was defined as a luminal diameter stenosis of ≥50% in any segment ≥1.5 mm in diameter. Although these patients had suspected coronary artery disease and underwent CTCA, only 8106 reported non-anginal chest pain, atypical angina, or typical anginal symptoms; this was the subgroup included in the pooled analysis. Foldyna *et al.*^3^ consisted of 4415 patients from the CT cohort of the randomized PROMISE trial, which tested functional testing against CTCA in symptomatic outpatients in whom physicians felt diagnostic testing for coronary artery disease was indicated.^13^ One-quarter of patients did not have chest pain as the presenting symptom, and of those that did, only 12% had typical angina. This study defined obstructive coronary disease as a diameter stenosis of ≥50% in any vessel with a calibre >2 mm. All recruiting centres in PROMISE were in North America. Reeh *et al.*^4^ retrospectively assessed 3903 patients who underwent invasive angiography at a single Danish centre for investigation of suspected angina. Most patients had already undergone at least one other test prior to angiography. In this study, obstructive disease was defined as a lesion requiring revascularization, diameter stenosis >70% or a fractional flow reserve <0.80. In contrast to these populations, the SCOT-HEART trial was designed as a pragmatic randomized trial and recruited a broader and less selected population of patients with suspected stable angina. Despite some differences in the pooled derivation population and the method used to diagnose obstructive coronary artery disease, we have shown a broadly similar distribution of ESC pre-test probabilities and observed disease prevalence in SCOT-HEART. Indeed, the highest pre-test probability—in males >70 years with typical angina—was 52% in the guideline cohort, correlating with an observed prevalence of 62% in SCOT-HEART. Importantly, all patients with a pre-test probability >15% belonged to groups that had a corresponding observed prevalence of >15%, while 8% of the cohort had a pre-test probability of 5–15% but belonged to groups that had a prevalence of >15%. This provides further evidence demonstrating the overestimation of previous pre-test probabilities, while highlighting differences between the ESC derivation populations and SCOT-HEART, where we found that the pre-test probability underestimated prevalence in some patients. We believe that this likely to reflect differences in the populations studied, with SCOT-HEART including a broader risk spectrum of patients as we have reported previously.^14^

In our current analysis, it would appear that less than half of patients referred with stable chest pain to the cardiology clinic will need further testing. Importantly, a large proportion of women will avoid investigations. Only women >60 years with typical angina or women >70 years with atypical angina have a pre-test probability >15%. In our cohort, an observed prevalence >15% was found in women >50 years with typical angina and women >70 years with any chest pain. These findings are important in light of recent gender disparities seen in SCOT-HEART, where women had proportionally more normal coronary arteries and less obstructive disease than men.^15^ Adoption of the latest guidelines may, therefore, result in less unnecessary investigations in women. Importantly, recent data have challenged the historic notion that women with myocardial infarction present more atypically than men.^16^ Although in SCOT-HEART there were relatively more women recruited with atypical chest pain, the low observed prevalence of disease in these groups confirms the importance of assessing symptoms on their own merit, independent of gender.

The concept of what constitutes low, intermediate, and high pre-test probability remains open for debate and is, to a degree, subjective. The previous 2013 ESC guidelines suggested that a pre-test probability of 15–85% constituted intermediate likelihood and further testing was warranted in the majority of patients.^12^ The 2019 ESC guidelines suggest that deferral of routine testing is safe in those with a pre-test probability of <15%, although discretionary testing if pre-test probability is 5–15% can be considered. However, pre-test probabilities for each patient group have been reduced, in recognition of prior overestimates of disease. Consequently, a much greater proportion of patients may be safely managed without further investigation, thus avoiding unnecessary tests in a group of patients with a low risk of adverse outcome (annual risk of cardiovascular death or myocardial infarction <1%).^5^ Our findings support this more conservative approach, with similarly low event rates in the <5% and 5–15% pre-test probability groups. Thus, reserving further investigation for those with a pre-test probability >15% may be an appropriate strategy. There will, of course, be scenarios where testing may be warranted in the 5–15% or even <5% groups, with many possible factors to consider. The negative predictive value of CTCA means that for some patients, the reassurance of a normal CTCA has major value and can reduce unnecessary anxiety and treatments.^17^

When proceeding to further investigation, what imaging modality should be used? After accounting for local factors, such as availability and expertise, the ESC guidelines recommend functional testing when there is a higher likelihood of disease (previously >50% pre-test probability) and CTCA when there is a lower likelihood (previously 15–50%).^5^^,^^12^ A recent meta-analysis based on Diamond and Forrester pre-test probabilities demonstrated CTCA to be most accurate in patients with a pre-test probability between 7% and 67%.^18^ A specific threshold is not stated in the latest guidelines but applying a 50% threshold to the current pre-test probabilities would infer that nearly all patients should have CTCA where possible. The recommendation for functional testing in patients with >50% pre-test probability is largely based on the recognized potential for discordance between anatomical stenosis severity and the haemodynamic effects of a lesion.^19^ Functional stress testing may provide valuable information about ischaemia in myocardial segments, as well as potential information about the patient’s functional capacity, which in itself is related to prognosis.^20^ However, functional testing does not provide anatomical information at a vessel or lesion level, nor does it provide adjunctive information regarding overall plaque burden. In this study, we found that within the >15% pre-test probability group, there appeared to be a similar proportionate reduction in the rate of fatal or non-fatal myocardial infarction in those above and below the median pre-test probability in those who underwent CTCA. This is an exploratory finding only, and we did not demonstrate a significant interaction between pre-test probability and the use of CTCA on multivariable analysis, but it highlights the need to balance the strengths and weaknesses of various available imaging modalities, as well as the intended purpose of the test: to help diagnose and manage symptoms of suspected angina due to obstructive coronary artery or prevent future myocardial infarction. Regardless of the imaging test chosen, studies have demonstrated a reduction in the rate of non-obstructive coronary artery disease on invasive angiography when an imaging test is performed beforehand,^13^^,^^21^^,^^22^ although CTCA is associated with the lowest rates of normal invasive angiography.

Perhaps not surprisingly, pre-test probabilities also track with future risk of events given that the presence and extent of obstructive coronary artery disease is a powerful predictor of future risk of adverse coronary events. Non-obstructive and obstructive disease increases across pre-test probability categories, and it is widely recognized that non-obstructive plaque and overall atherosclerotic burden strongly correlate with outcomes.^23–25^

This study is a *post hoc* subgroup analysis and the results are hypothesis-generating only. Although our findings are largely consistent with contemporary pre-test probability estimates, the observed prevalence within each category of age band, sex, and chest pain type is based on relatively small numbers. In particular, the cohort is underpowered to conclusively demonstrate the benefit of CTCA within subgroups of pre-test probability (particularly the lower two strata) with regards to clinical outcomes. Further data to confirm the benefits of discretionary testing in patients with a pre-test probability of 5–15% would be of substantial interest. We did not include dyspnoea as a symptom in SCOT-HEART and were thus unable to validate the ESC pre-test probabilities for this group. However, our main findings are congruent with the large existing body of literature that has demonstrated the overdiagnosis of coronary artery disease when utilizing previous pre-test probability models. Our findings are also in keeping with the data upon which the guidelines are based, promoting a more conservative approach to the use of downstream investigations in patients with suspected stable angina, and demonstrating that the greatest benefits of CTCA appears to be in those with a pre-test probability >15%.^26^

## Conclusion

The pre-test probabilities for obstructive coronary artery disease recommended by the 2019 ESC chronic coronary syndrome guidelines are broadly consistent with the SCOT-HEART trial cohort, although prevalence is underestimated in some groups. Patients with a high pre-test probability had a worse prognosis and this group had the greatest observed numerical difference in events with CTCA compared to standard care. These findings provide strong support for the recommended strategy of offering non-invasive imaging to patients with a pre-test probability of >15%.

## Supplementary material

Supplementary material is available at *European Heart Journal – Quality of Care and Clinical Outcomes* online.

## Funding

This work was supported by the Chief Scientist Office of the Scottish Government (CZH/4/588, PCL/17/04 to M.W.), the British Heart Foundation (CH/09/002, RE/13/3/30183, RE/18/5/34216, PG/19/40/34422, and RG/16/10/32375 to D.E.N., FS/11/014 to M.W., FS/16/14/32023 to N.L.M., and FS/14/78/31020 to M.R.D.), the National Heart Foundation of New Zealand (1844 to P.D.A.), the Wellcome Trust (WT103782AIA to D.E.N.), and National Health Service Research Scotland.

**Conflict of interest:** none declared.

## Supplementary Material

qcaa006_supplementary_data
