# Supplementary material for: Validation of European Society of Cardiology pre-test probabilities for obstructive coronary artery disease in suspected stable angina
Source: Eur Heart J Qual Care Clin Outcomes. 2020 Jan 24;6(4):293–300. doi: 10.1093/ehjqcco/qcaa006 (PMC7590886; doi:10.1093/ehjqcco/qcaa006)
Supplement: qcaa006_supplementary_data [file qcaa006_supplementary_data.zip › supp_tables.docx]

| **Supplementary Table 1** Therapies for pre-test probability groups by study allocation | | | | | | |
| --- | --- | --- | --- | --- | --- | --- |
|  | **<5%** | | **5-15%** | | **>15%** | |
|  | **Standard** | **CTCA** | **Standard** | **CTCA** | **Standard** | **CTCA** |
| **n** | 434 | 397 | 638 | 664 | 807 | 815 |
| **Antiplatelet - new** | 4 (0.9) | 29 (7.3) | 13 (2.0) | 94 (14.2) | 16 (2.0) | 94 (11.5) |
| **Antiplatelet - stopped** | 0 (0.0) | 4 (1.0) | 1 (0.2) | 40 (6.0) | 3 (0.4) | 29 (3.6) |
| **Statin - new** | 6 (1.4) | 29 (7.3) | 26 (4.1) | 99 (14.9) | 48 (5.9) | 95 (11.7) |
| **Statin - stopped** | 0 (0.0) | 3 (0.8) | 2 (0.3) | 17 (2.6) | 4 (0.5) | 15 (1.8) |
| **Invasive angiography** |  |  |  |  |  |  |
| - **Overall** | 17 (3.9) | 20 (5.0) | 93 (14.6) | 84 (12.7) | 314 (38.9) | 306 (37.5) |
| - **1 year** | 5 (1.2) | 14 (3.5) | 73 (11.4) | 59 (8.9) | 265 (32.8) | 280 (34.4) |
| **PCI** |  |  |  |  |  |  |
| - **Overall** | 9 (2.1) | 6 (1.5) | 26 (4.1) | 25 (3.8) | 144 (17.8) | 152 (18.7) |
| - **1 year** | 1 (0.2) | 4 (1.0) | 19 (3.0) | 21 (3.2) | 119 (14.7) | 135 (16.6) |
| **CABG** |  |  |  |  |  |  |
| - **Overall** | 0 (0.0) | 1 (0.3) | 1 (0.2) | 3 (0.5) | 46 (5.7) | 57 (7.0) |
| - **1 year** | 0 (0.0) | 1 (0.3) | 1 (0.2) | 1 (0.2) | 38 (4.7) | 52 (6.4) |
| Abbreviations: CTCA, computed tomography coronary angiography; PCI, percutaneous coronary intervention; CABG, coronary artery bypass grafting | | | | | | |

|  |  |  | |  |  | |  | |  |
| --- | --- | --- | --- | --- | --- | --- | --- | --- | --- |
| **Supplementary Table 2** Clinical outcomes in patients with pre-test probability >15%, stratified above and below median. | | | | | | | | | |
| **Pre-test probability** | | | **Standard care** | | | **CTCA** | | **p** | |
| **≥ 26%** | | | 415 | | | 450 | |  | |
| NFMI/CHD death | | | 26 (6.2) | | | 16 (3.6) | | 0.06 | |
|  | | |  | | |  | |  | |
| **16-26%** | | | 392 | | | 365 | |  | |
| NFMI/CHD death | | | 17 (4.3) | | | 7 (1.9) | | 0.057 | |
| Abbreviations: CTCA, computed tomography coronary angiography; NFMI, non-fatal myocardial infarction; CHD, coronary heart disease | | | | | | | | | |

| **Supplementary Table 3** Cox regression models for non-fatal myocardial infarction or coronary heart disease death | | | | | | |
| --- | --- | --- | --- | --- | --- | --- |
|  | **Univariable analysis** | | | **Multivariable analysis** | | |
|  | **HR** | **95% CI** | **p value** | **HR** | **95% CI** | **p value** |
| **ESC PTP** |  |  |  |  |  |  |
| **5-15%** | 1.02 | 0.50-2.11 | 0.95 | 0.93 | 0.38-2.32 | 0.88 |
| **>15%** | 2.88 | 1.56-5.33 | <0.001 | 2.91 | 1.37-6.20 | 0.006 |
| **Study allocation - CTCA** | 0.56 | 0.37-0.85 | 0.006 | 0.55 | 0.17-1.82 | 0.33 |
| **BMI ≥30** | 0.93 | 0.62-1.40 | 0.72 | 0.93 | 0.61-1.41 | 0.73 |
| **Diabetes mellitus** | 0.59 | 0.97-2.92 | 0.06 | 1.59 | 0.91-2.79 | 0.11 |
| **Atrial fibrillation** | 0.51 | 0.07-3.67 | 0.50 | 0.43 | 0.06-3.11 | 0.41 |
| **ESC PTP 5-15% * CTCA** |  | | | 1.27 | 0.28-5.74 | 0.75 |
| **ESC PTP >15% * CTCA** |  |  |  | 0.96 | 0.26-3.52 | 0.95 |
| Abbreviations: ESC, European Society of Cardiology; HR, hazard ratio; CI, confidence interval; CTCA, computed tomography coronary angiography; BMI, body mass index.  * Denotes interaction term | | | | | | |
