# Supplementary figures and images for: Validation of European Society of Cardiology pre-test probabilities for obstructive coronary artery disease in suspected stable angina
Source: Eur Heart J Qual Care Clin Outcomes. 2020 Jan 24;6(4):293–300. doi: 10.1093/ehjqcco/qcaa006 (PMC7590886; doi:10.1093/ehjqcco/qcaa006)

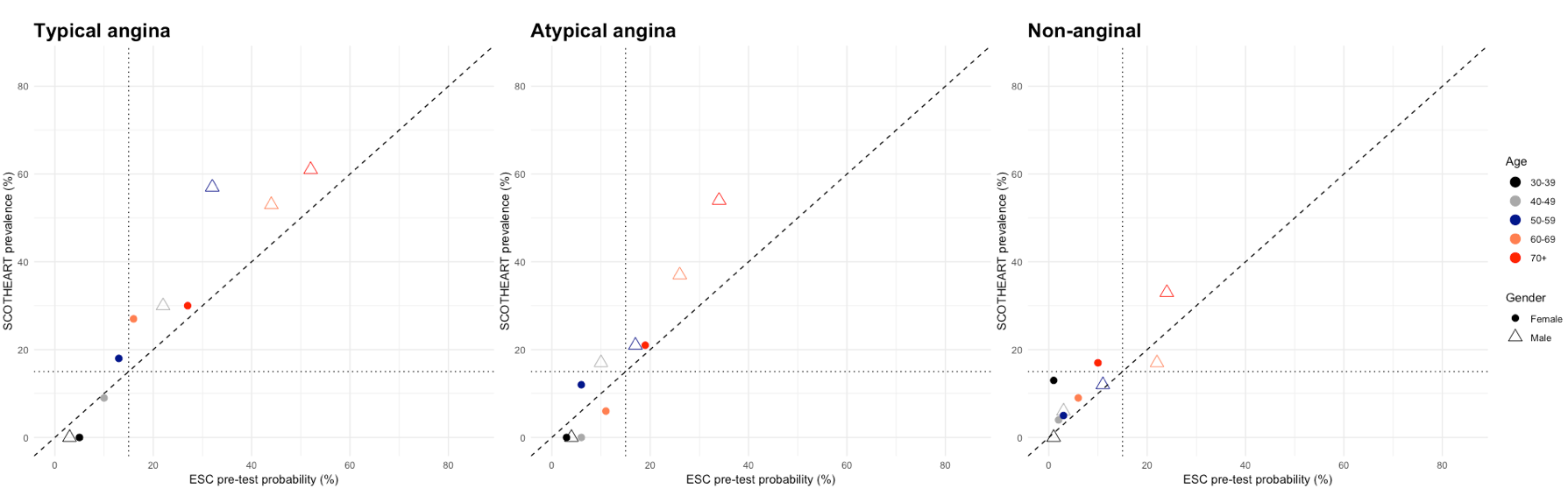

Supplement: qcaa006_supplementary_data [file qcaa006_supplementary_data.zip › supp_fig_2.tiff]

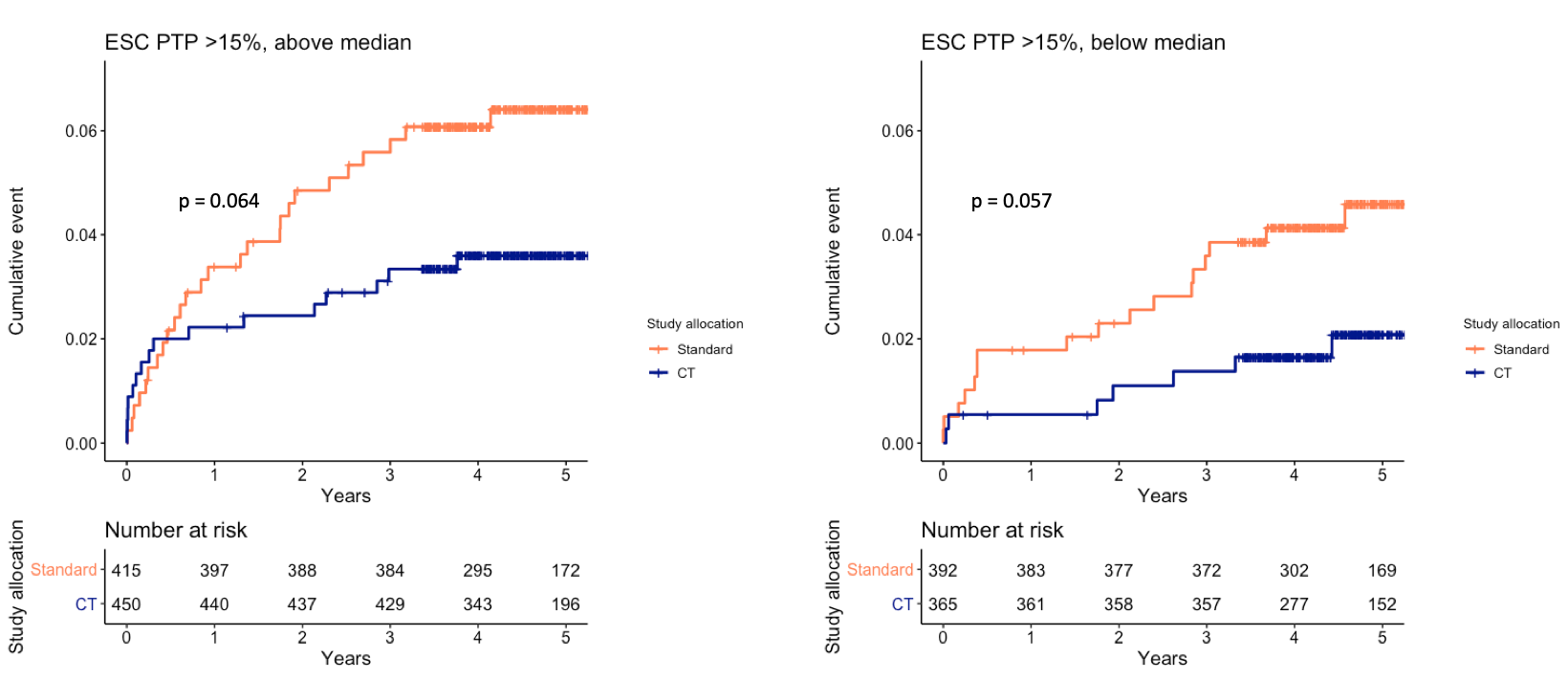

Supplement: qcaa006_supplementary_data [file qcaa006_supplementary_data.zip › supp_fig_3.tiff]

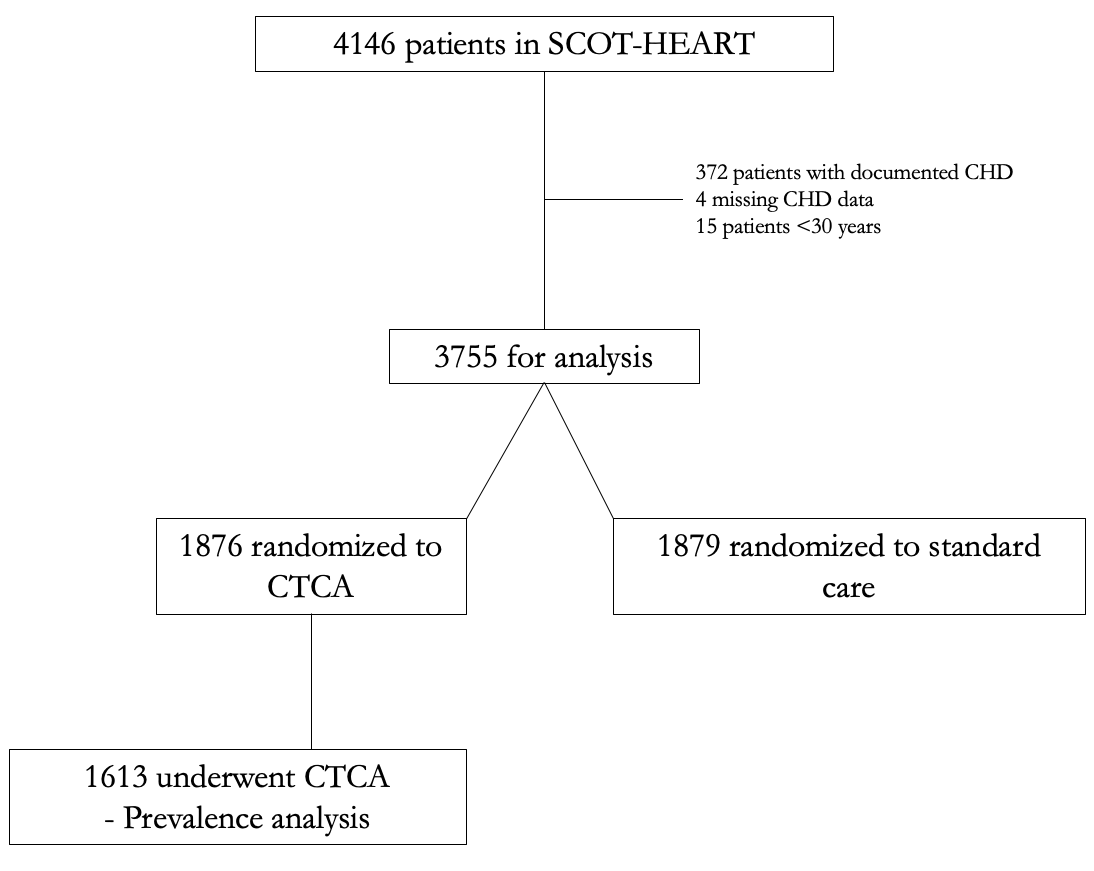

Supplement: qcaa006_supplementary_data [file qcaa006_supplementary_data.zip › supp_fig_1.tiff]
